# Comprehensive Cardiovascular Management of Myotonic Dystrophy Type 1 Patients: A Report from the Italian Neuro-Cardiology Network

**DOI:** 10.3390/jcdd11020063

**Published:** 2024-02-16

**Authors:** Vincenzo Russo, Giovanni Antonini, Roberto Massa, Carlo Casali, Alfredo Mauriello, Anna Maria Martino, Roberto Marconi, Matteo Garibaldi, Pasquale Franciosa, Massimo Zecchin, Carlo Gaudio, Antonello D’Andrea, Stefano Strano

**Affiliations:** 1 Cardiology Unit, Department of Medical Translational Sciences, University of Campania “Luigi Vanvitelli”—“Monaldi” Hospital, 80126 Naples, Italy; alfredo.mauriello93@libero.it; 2 Neuromuscular Disease Centre, Department of Neurosciences, Mental Health and Sensory Organs (NESMOS), Sapienza University of Rome, “Sant’Andrea” Hospital, Via di Grottarossa, 1035-1039, 00189 Rome, Italy; giovanni.antonini@uniroma1.it (G.A.); matteo.garibaldi@uniroma1.it (M.G.); 3 Neuromuscular Diseases Unit, Department of Systems Medicine, University of Rome “Tor Vergata”, 00133 Rome, Italy; massa@uniroma2.it; 4 Department of Medico-Surgical Sciences and Biotechnologies, Sapienza University of Rome, 00196 Rome, Italy; carlo.casali@uniroma1.it; 5 Department of Cardiology, “Umberto I” Hospital, 84014 Nocera Inferiore, Italy; antonellodandrea@libero.it; 6 Division of Cardiology, Policlinico Casilino, 00169 Rome, Italy; annamaria.martino@hotmail.it; 7 Unit of Neurology, Cardio-Thoracic-Neuro-Vascular Department, “Misericordia” Hospital, 58100 Grosseto, Italy; roberto.marconi@uslsudest.toscana.it; 8 Department of Internal, Anesthesiological and Cardiovascular Clinical Sciences, Sapienza University of Rome, 00196 Rome, Italy; pasquale.franciosa@uniroma1.it (P.F.); carlo.gaudio@uniroma1.it (C.G.); stefano.strano@uniroma1.it (S.S.); 9 Cardiothoracovascular Department, “Cattinara” Hospital, ASUGI and University of Trieste, 34149 Trieste, Italy; massimo.zecchin@asugi.sanita.fvg.it

**Keywords:** myotonic dystrophy, cardiovascular disease, neurological disease, sudden cardiac death, arrythmogenic risk

## Abstract

Myotonic dystrophy is a hereditary disorder with systemic involvement. The Italian Neuro-Cardiology Network-“Rete delle Neurocardiologie” (INCN-RNC) is a unique collaborative experience involving neurology units combined with cardio-arrhythmology units. The INCN facilitates the creation of integrated neuro-cardiac teams in Neuromuscular Disease Centers for the management of cardiovascular involvement in the treatment of myotonic dystrophy type 1 (MD1).

## 1. Introduction

The Italian Neuro-Cardiology Network-“Rete delle Neurocardiologie” (INCN-RNC) is a unique collaborative experience involving neurology units paired with cardio-arrhythmology unit. In January 2021 the 8th INCN-RNC annual meeting raised the need for a coordinated and integrated model of care for patients with MD1. The board of neuromuscular disease experts discussed the current and emerging apparent gaps in the comprehensive care of cardiovascular involvement in MD1, including operational/logistical issues for health systems and integrated networks, to reach a consensus. Cardiovascular comorbidities in the MD1, despite substantial advances in research and clinical care, have relevant gaps in clinical evidence and cause uncertainty about best practices for the treatment and early diagnosis of arrhythmic and non-arrhythmic disorders. On January 2021 and May 2022 at the annual National meetings of INCN-RNC, two symposia and round tables on “The practical management of cardiac involvement in patients with myotonic dystrophy: the need for interdisciplinary action” took place. On February 2023, during the 10th annual meeting of the INCN-RNC, the council approved the first draft of this consensus document; on December 2023 the following definitive version was approved.

### Myotonic Dystrophy

Myotonic dystrophy (MD) is a dominantly inherited multisystem disorder caused by expanded CTG repeats in the 3′ UTR of the *DMPK* gene (MD1) or CCTG repeats in the first intron of the *CNBP* gene (myotonic dystrophy type 2 or MD2). The main pathogenic mechanism of MD is the toxic gain of function of RNAs transcribed from expanded alleles that fold into hairpin structures and accumulate in nuclear foci, interfering with the activity of muscle-blind-like (MBNLs) and CUGBP Elav-like Family Member 1 (CELF1) proteins [1]. These two classes of RNA binding proteins antagonistically regulate the alternative splicing of developmental genes, and their alterations in MD tissues (skeletal muscle, heart, brain, etc.) lead to an aberrant alternative splicing of multiple genes, with the preferential expression of immature protein isoforms [2]. Mouse models have been used to evaluate the RNA sequence that determines the most severe forms of MD1. Furthermore, several therapeutic approaches have been developed which are based on the use of viral vectors and antisense oligonucleotides (ASOs), providing a promising approach to the treatment of myotonic dystrophy type 1 because they reduce toxic RNA levels [3]. Despite many clinical and molecular similarities, MD1 and MD2 manifest with different features, including a lack of congenital or childhood forms, minimal facial signs, and reduced risk of cardiac conduction defects in MD2 compared to MD1, together with different distribution of muscle involvement and the pattern of muscle involvement in muscle biopsy and muscle MRI between the two forms [4]. This issue could be explained by the concurrence of other pathogenic mechanisms able to modulate the phenotype, including epigenetic modifications at the respective gene loci induced by the pathological expansions, the occurrence of antisense “RNA” translation, and haploinsufficiency of the respective genes [5]. 

MD, as a whole, is a common type of muscular dystrophy among adult Caucasians. However, the geographic and ethnic distribution of this disease is very uneven among different populations. Prevalence estimates of molecularly defined MD1 report values spanning over a very large range, between 0.43 and 158 cases per 100.000, depending on the population studied. This enormous variability reflects a very low prevalence of the disease in the Far East, as opposed to very high rates, depending on founder effects, observed among French Canadians, Basques, and Afrikaners. On the other hand, MDs are virtually absent in native populations of the Americas, Africa, and Oceania [6]. A recent study performed in the province of Rome, Italy, provided, for the first time, age-adjusted, sex- and age-specific prevalence estimates of MD1 and MD2 in the same area, with values of 8.35/100,000 for females and 11.07/100,000 for males in MD1. The incidence of MD1 is 20 cases per million person-years. Values about tenfold lower were reported for MD2, with a slight female preponderance [6]. MD1 and MD2 are progressive, multisystem disorders, characterized by muscular, cardiovascular, respiratory, and cognitive impairment. The onset of symptoms can be at different ages, but generally, symptoms appear in the second or third decade of life [7,8,9]. In addition, several features of the metabolic syndrome are common, including insulin resistance, increased waist circumference, dyslipidemia, and reduced levels of adiponectin. Surprisingly, despite the presence of all these metabolic risk factors, MD1 patients do not have higher chances of developing diabetes mellitus, coronary heart disease, or stroke, compared to the general population. On the other hand, MD1 is associated with an increased risk of developing several types of benign or malignant tumors [10]. At present, there is no curative or disease-modifying treatment for MD1 or MD2, and management focuses on genetic counseling, preserving function and independence, preventing cardiopulmonary complications, and symptomatic treatment of myotonia, daytime sleepiness, etc. [11,12].

## 2. Cardiovascular Involvement in MD1

Cardiac involvement occurs in 80% of MD1 patients and it often precedes the involvement of skeletal muscle [13]. Cardiac involvement in patients with MD1 occurs as a degenerative process, with progressive fibrosis and fatty replacement of the myocardium, which involves not only the specialized conduction system but also areas, initially unaffected, of the atrial and ventricular myocardium [14,15,16]. This anatomy-pathologic substrate may, on the one hand, facilitate the development of cardiac conduction diseases, ventricular tachycardia (VT), and sudden cardiac death (SCD) on the other hand, it may be responsible for ventricular dyssynchrony, leading to cardiomyopathy with systolic dysfunction [7,17].

### 2.1. Conduction System Disease

An impairment of the conduction system is common cardiac abnormality in MD1 patients. First-degree atrioventricular block (AVB) (28.2–34.1%) and QRS complex >120 ms (18.4–19.9%) are the most frequent abnormalities found [18,19]. These abnormalities identifies a subgroup of MD1 patients in need of cardiac pacing, because they are considered independent predictors of a prolonged His-ventricle (HV) interval ≥70 ms in electrophysiological study (EPS) [20,21]. 

### 2.2. Atrial Fibrillation (AF)

AF has a prevalence of 11% in MD1 patients, about 70-fold higher than the general population [22]. If we consider cardiac-implanted electronic device-detected AF events, this value increases to 25% [23,24,25]. There are several risk factors which have been identified as predictors of AF development: male sex, low left ventricular ejection fraction (LVEF), electro-mechanical echocardiographic and electrocardiographic abnormalities [22,26]. MD1 patients with AF have higher overall mortality than those without AF [22]; however, the association with SCD is still controversial. Therefore, non-sustained supraventricular tachycardia is reported with a prevalence of 37%.

### 2.3. Ventricular Arrhythmias

The prevalence rates of non-sustained and sustained VT were 2.2% and 0.8%, respectively [27]. In MD1 patients who are in need of permanent cardiac pacing, a previous episode of non-sustained VT is considered the only independent predictor of sustained VT [19]; therefore, it is used as a criterion for the preference of an implanted cardioverter defibrillator (ICD) over a pacemaker (PM) [28,29]. For this reason, the early identification of a non-sustained VT in MD1 patients is an aim in the management and prevention of SCD [30]. Several studies have shown evidence of increased dispersion of ventricular repolarization (QTc dispersion, JTc dispersion, transmural dispersion of repolarization, QT variability index) and sympathovagal balance in patients with MD1 (heart rate variability) suggesting the potential interest of these measures to predict ventricular arrhythmias [31,32,33,34].

### 2.4. Sudden Cardiac Death

Pneumonia and cardiac arrhythmias are the most frequent primary causes of death. [35] SCD has an annual incidence of 0.53–1.16% [36,37], three-fold higher in MD1 patients than in age-matched healthy controls. Even if the mechanisms leading to SCD remain controversial, extreme bradycardia sch as the complete AVB, asystole, and VT may represent the most prevalent cause of SCD in MD1 patients [38].

Independent predictors of SCD are (i) clinical diagnosis of atrial tachyarrhythmia and electrocardiogram (ECG) with one of the following features: any rhythm other than sinus rhythm, PR interval ≥ 240 ms, QRS duration ≥ 120 ms, second- or third-degree atrioventricular block [37,39], age, family history of SCD, and left bundle branch block [40,41].

### 2.5. Cardiomyopathy and Heart Failure

Different from arrhythmias, little is still known about the epidemiology of left ventricular (LV) dysfunction and heart failure (HF) among MD1 patients [42]. The prevalence of LV systolic dysfunction (LVSD) (EF < 54%), assessed by trans-thoracic echocardiography (TTE), ranged from 0% to approximately 21% [42]. The causes of LVSD are not completely understood; however, they might include intra-ventricular (IV) and atrioventricular (AV) conduction time delay, atrial or ventricular arrhythmias, and ventricular myocardial fibrosis, until a dilatated cardiomyopathy. MD1 patients with prolonged PR or QRS intervals showed a four times higher risk of developing LVSD or HF [43,44]. Among MD1 patients with AF, the prevalence of LVSD accounted for up to 46% [42]. Contrast-enhanced cardiac magnetic resonance imaging (MRI) studies have detected myocardial fibrosis in 13–40% of MD1 patients [45,46,47].

Data regarding LV diastolic dysfunction (LVDD) in MD1 patients are quite lacking. To date, mild diastolic dysfunction has been observed in 5–50% of MD1 patients [48]. The diastolic dysfunction in MD1 might be related to AF, fibrotic degenerative changes in the myocardium (likely affecting LV relaxation), and impaired calcium metabolism in cardiomyocytes. No association between LVDD and AV or IV conduction defects has been observed [49]. Whether AV/IV conduction defect may cause LV mechanical impairment or whether both electrical and mechanical impairments may be the common result of fibrosis of the myocardium and conduction system still needs to be clarified. 

The prevalence of symptomatic HF in MD1 subjects ranges from 0% to 7.1% [37]; however, HF symptoms should be underestimated due to the limited level of activity of MD1 patients. The early diagnosis of HF disease is of pivotal importance since it increases the risk of all-cause death by four times, and the risk of cardiac death by six times [44]. 

Therefore, because there are no trials which demonstrate any benefits, it is reasonable that treatment for HF should be started early. In particular, angiotensin-converting enzyme (ACE) inhibitors and angiotensin II receptor antagonists (ARB) could be of particular benefit in MD1 for anti-fibrotic properties [50] with LVEF < 50%. Beta-blockers should be reserved for patients without AV conduction abnormalities or recipients of PM and/or ICD; the up-titrate drug dosage should be applied based on individual response and toleration. Cardiac resynchronization therapy is recommended for patients with persistent symptomatic HF (New York Heart Association functional class III) due to LVSD (LVEF < 35%) with large QRS (>150 ms) with left bundle branch block pattern and a normal sinus rhythm while receiving optimal guideline-directed medical therapy [51,52].

### 2.6. Hypotension

It is generally recognized by clinicians that MD1 subjects have low blood pressure (BP) values. However, only a few non-systematic studies have shown that consecutive MD1 patients have significantly lower BP values than healthy control subjects [53]. It is still not clear whether low BP may be related to the pathophysiology of the disease or the autonomic cardiac dysfunction, predominantly parasympathetic, that is common in MD1 subjects, or if it may be a specific complication of the disease related to the genetic mutation [54]. However, low BP values have recently been demonstrated to be a marker of disease severity and to contribute, when added to other clinical, electrocardiographic, and respiratory parameters, to stratifying MD1 patients at risk of death [55].

### 2.7. Stroke and Systemic Embolism

The prevalence of both symptomatic and asymptomatic ischemic strokes in MD1 patients was about 6.5% [56]. The AF/flutter was found in 55% of MD1 patients with ischemic stroke. All patients with stroke had CHADS2 [Congestive heart failure, Hypertension, Age ≥ 75 years, Diabetes mellitus, prior Stroke or TIA or thromboembolism (doubled)] and CHA2DS2-VASc [Congestive heart failure, Hypertension, Age ≥ 75 years (doubled), Diabetes mellitus, prior Stroke or TIA or thromboembolism (doubled), Vascular disease, Age 65 to 74 years, Sex category; HF: heart failure; TIA: transient ischemic attack; TE: thromboembolism; MI: myocardial infarction; PAD: peripheral artery disease] scores higher than two [57]. An expert consensus opinion of the AHA for the management of MD1 patients suggests the use of the CHA2DS2-VASc score to stratify thromboembolic risk; however, it also outlines the need to carefully consider their increased fall risk due to underlying neuromuscular disease and muscle weakness. Since studies comparing vitamin K antagonists (VKAs) and direct oral anticoagulants (DOACs) in this clinical setting are lacking, a careful evaluation of renal function is warranted, eventually based on the dosage of cystatin C, because serum creatinine may be low to non-detectable in a setting of low muscle mass (which is not uncommon in MD1 subjects) [51,58].

## 3. Non-Invasive Cardiac Evaluation

To optimize the clinical management of MD1 patients, neurologists should identify referent cardiologists/electrophysiologists with expertise in neuromuscular disorders early [51].

Cardiologic evaluation is comprehensive of ECG, TTE, and 24-h Holter ECG monitoring and is highly recommended at the time of disease diagnosis. A cardiologic clinical history investigation should focus on eventual warning symptoms, including healing, dizziness, pre-syncope, syncope, or breathlessness. Moreover, even for completely asymptomatic subjects, annual cardiologic visits with ECG are recommended, since MD1 is a progressive disease [52]. Given the increased prevalence of AF in MD1 patients and its association with higher overall mortality, we suggest performing careful electrocardiographic monitoring by 24-h Holter ECG at least annually in the overall MD1 population and daily remote monitoring (RM) of those with cardiac implantable electronic devices (CIEDs). For MD1 patients at increased risk of AF, according to electrocardiographic and echocardiographic risk parameters, an external loop recorder should be considered. 

### 3.1. Twelve Lead ECG

Twelve-lead ECG is an essential tool for risk stratification of life-threatening arrhythmic disorders in MD1 patients. It is indicated in all patients upon confirmation of MD1 diagnosis, and annually thereafter, due to the risk of disease progression [51,52]. A PR interval > 200 ms and/or QRS duration > 100 ms should be an indication of the need to perform an EPS for detecting a prolonged HV interval (>70 ms) in need of cardiac pacing [20,59,60]. However, it should be noted that up to 66.1% of MD1 patients with these electrocardiographic findings may have normal HV intervals [61]. Moreover, in MD1 patients with QRS > 120 ms and PR > 240 ms, a pacemaker may be considered to reduce the risk of SCD [29,37].

### 3.2. 24 h Holter ECG Monitoring

Ambulatory electrocardiographic monitoring is a useful tool for the identification of paroxysmal second or third-degree AV blocks, or intermittent bundle branch blocks, that do not appear at rest ECG. It is indicated at the time of MD1 diagnosis and in the case of the occurrence of either ECG abnormalities (AV or intra-ventricular blocks) or symptoms including heeling, dizziness, pre-syncope, and syncope [52]. Moreover, it may be useful to identify asymptomatic episodes of non-sustained VT or paroxysmal AF, which may impact the patients’ prognosis and need careful management [19,62].

### 3.3. Transthoracic Echocardiogram

TTE is the most widely used imaging tool to obtain structural and functional information about the heart. MD1 patients should undergo a cardiac imaging examination at baseline and every 1 to 5 years thereafter if the initial imaging study is normal [52]. Particular attention should be given to subjects with baseline electrocardiographic abnormalities or arrhythmias, since systolic dysfunction seems to be more common in these subgroups [49]. Moreover, new echocardiographic techniques, such as three-dimensional (3D) TTE or speckle tracking analysis, should be performed to empower bi-dimensional TTE diagnostic and prognostic ability [48].

### 3.4. Cardiac Magnetic Resonance

Contrast-enhanced cardiac MRI is a highly sensitive, non-invasive tool for the detection of functional and structural myocardial abnormalities. Besides parameters easily available with TTE examination, cardiac MRI may detect eventual myocardial damage suggestive of scarring through late gadolinium enhancement (LGE) [63]. Moreover, it may quantify interstitial fibrosis through the extracellular volume (ECV) fraction technique [64] and can detect even subtle myocardial deformation or contractility impairment (as per localized degenerated myocardial tissue) through the cardiac strain technique [65].

Several observational MRI studies showed cardiac structural abnormalities among MD1 subjects, including reduced LV [47,66] or right ventricle [46] systolic function, LV hypertrophy [47] and LV non-compaction [46,67,68]; moreover, reduced values of myocardial strain, both in the longitudinal, circumferential and strain area components, have been described among MD1 patients with preserved LVEF, as per an early detection of LV contractility impairment [66,69]. (Interestingly, a non-negligible prevalence of LV LGE with a non-ischemic distribution pattern (i.e., in the mid-wall or subepicardial myocardial layers), mostly located in the inter-ventricular septum or the postero-lateral wall, has been detected in MD1 subjects, with a prevalence ranging from 12.5% [46,47] to 42% [70,71,72]. The prognostic role of LGE or interstitial fibrosis in MD1 patients is still debated and needs of further studies [72,73].

## 4. Invasive Cardiac Evaluation and Treatment

### 4.1. Electrophysiological Study

EPS should increase the accuracy of SCD risk stratification in MD1 patients with electrocardiographic abnormalities (PR interval > 200 ms or QRS > 100 ms) through the evaluation of HV interval prolongation (>70 ms), which identifies those in need of prophylactic cardiac pacing. To date, little is known about the timing and the role of programmed ventricular stimulation for arrhythmic risk stratification [30,74]. The ACADEMY 1, a prospective single-center study about the electrophysiological study-guided ICD strategy in the prevention of arrhythmic cardiac death in MD1 patients, suggests the inducibility of VT has a limited value in the arrhythmic risk stratification among MD1 patients [75,76].

### 4.2. Loop Recorder

An implanted loop recorder (ILR) should be considered as an option for detecting clinically asymptomatic conduction disorders or spontaneous VT and for helping in the decision about the best choice of device to prevent SCD. It should be useful in MD1 patients with first-degree AV, fascicular, or bundle branch block and HV interval < 70 ms [77].

### 4.3. Pacemaker

Permanent cardiac pacing is indicated in patients with any second- and third-degree AVB or His-ventricle (HV) interval > 70 ms, regardless of the symptoms, and it may be considered in those with QRS > 120 ms and PR > 240 ms. Atrial pacing in the Bachmann bundle region was associated with a reduction in atrial electromechanical delay and the risk of R-wave oversensing on the atrial lead [78], compared with right atrial stimulation; however, it showed no benefit for the prevention of AF onset [79]. The activation of right atrial preference pacing [80] and minimal ventricular pacing [81] algorithms seems to be an efficient strategy to reduce the risk of AF in MD1 patients implanted with a PM. An increase in the incidence of AF has been shown in patients with a higher rate of right ventricular pacing and a lower rate of atrial stimulation [82].

### 4.4. Implantable Cardioverter-Defibrillator (ICD)

ICD implantation may be considered for all MD1 patients with permanent pacing indication and spontaneous or EPS inducible VT, regardless of their symptoms or LVEF. Cardiac resynchronization therapy (CRT) may be an option in MD1 patients with bundle branch block (especially left bundle branch block) who need permanent pacemaker implantation; however, there are currently only a few case reports about CRT therapy in MD1 patients [83,84].

### 4.5. CIED Remote Monitoring

RM combined with interrogation of the cardiac implanted device (PM, ICD, or ILR) and at least annual evaluation should be adopted to improve the clinical management of asymptomatic arrhythmias and to reduce the family-provided healthcare costs for MD1 patients with motor disability [85].

## 5. Open Issues/Improvement Areas in the Organization of Services

Although there is a consensus that cardiological assessments should generally be planning once a year, this remains arbitrary, as the lack of a reliable biological marker able to identify patients at higher cardiological risk does not allow for stratifying patients based on cardiological risk and, therefore, for establishing a risk-based cardiological assessment follow-up. In this regard, the usefulness of genetic data as a predictor of cardiac complications in MD1 is controversial. Whereas CTG expansion length correlates with the age at onset of cardiac complications in those patients showing cardiac rhythm or conduction abnormalities, it seems to not predict the occurrence of cardiac complications in MD1 [86]. A possible explanation of this leak correlation could be that CTG expansion is different among tissues and unstable over time. Possibly, CTG expansion detected on the leukocytes from blood sampling at the time of diagnosis could not reflect the expansion in myocardiocytes at the time of development of cardiac abnormalities, which could explain the weak correlation obtained in previous studies. Conversely, the male gender seems to be associated with a higher risk of cardiac complications in MD1, suggesting that in clinical assessment, MD1 male patients should be monitored with higher attention in the clinical follow-up for the occurrence or progression of cardiac involvement [87]. Possibly, the routine use of cardiac MRI or electrophysiological studies could help to better stratify the risk among patients, even if these methods are expensive or invasive and the timing of these studies is still controversial [60].

## 6. Disease Management Model: The “Neuro-Cardiac Team”

The comprehensive care of patients with neuromuscular diseases and those affected by MD1 is an interdisciplinary challenge. The close collaboration of cardiologists and neurologists with expertise in neuromuscular diseases is essential to ensure the optimal use of short- and long-term care and tests for the early diagnosis of cardiovascular involvement. This collaboration should be based on the cooperative model to share decision making tailored to clinical scenarios (Neuro-Cardiac Team). The INCN facilitates the establishment of Neuro-Cardiac Teams integrated into the Centers for Neuromuscular Diseases for the management of cardiovascular involvement in the treatment of MD1.

## 7. Quality Improvement and Risk Management

MD1 patients need development process, outcome, individual practitioner level, and system level quality measures, as seen in Table 1. These measures may be used to calculate performance at the practitioner level or system level. The greatest impact of the measures depends on their appropriate use and is linked directly to operational steps that clinicians, patients, and health plans can apply in practice to improve care [11,81]. However, performance measurement may not achieve the desired aim of improving patient care by itself.

The function of clinical risk management is essentially to provide the Neuro-Cardiac Team with the information necessary to “learn from errors” or preventable adverse events and so-called “near-events” or near-misses. The Neuro-Cardiac Team, for this purpose, must at first prepare and implement tools aimed at the qualitative/quantitative identification of risks and specific critical issues using a proactive approach. The proactive analysis starts from the assumption that errors can be prevented by investigating the processes at all stages and aims to identify system criticalities and possible areas of human error, to prevent them from occurring. Failure Mode Effects Analysis (FMEA) is a method that allows us to identify possible failure modes/errors, their effects, and potential causes. Failure Mode Effects and Criticality Analysis (FMECA) adds a quantitative analysis to the FMEA that allows for the classification of the Failure Modes/Errors based on an Index of Risk Priority (IPR). The numerical index (IPR) is constructed using scoring scales that consider the probability of the error occurring, the possibility of it being detected and the severity of its consequences. It is used in the application of the FMECA and defines the criticality level of a process. The value of the risk priority index helps to make decisions for the activation of prevention measures [88]. The application of the FMEA/FMECA consists of breaking down a process into individual tasks: the Neuro-Cardiac Team starts the analysis with the review of existing processes and procedures, identifying, in the various phases, the critical points. This approach can also be used in the conception and design of new procedures, processes, and technologies to create protective barriers that prevent human/active error in MD1 patients.

## 8. Integrated Interdisciplinary Comprehensive MD1 Pathway

**Figure 1:**
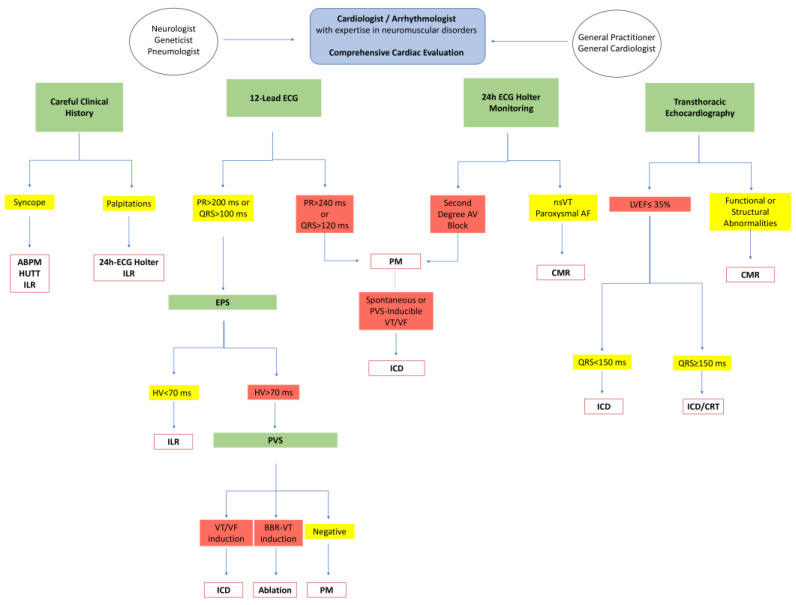
Flow chart of integrated interdisciplinary comprehensive MD1 pathway. ABPM: ambulatory blood pressure monitoring; AF: atrial fibrillation; BBR: bundle branch reentrant; CMR: cardiac magnetic resonance; CRT: cardiac resynchronization therapy ECG: electrocardiogram; ELR: external loop recorder; EPS: electrophysiological study; HUTT: head-up tilt test; ICD: implantable cardiovert defibrillator; ILR: implantable loop recorder; LVEF: left ventricular ejection fraction.; nsVT; non-sustained ventricular tachycardia; PM: pacemaker; PSV: programmed ventricular stimulation; VF: ventricular fibrillation; VT: ventricular tachycardia. Yellow: condition with mild morbidity. Red: condition with severe morbidity.

A strategy based on the stratification of cardiovascular risk must be implemented by the neurologist already in the initial evaluation phase of the patient, to be able to decide the level of complexity of cardiological investigations according to practical clinical paths shared with the reference cardiologist (Figure 1).

## 9. Perspective

Notably, forthcoming approaches to treat MD1 target the toxic mRNA product, aiming to reverse the pathophysiological mechanisms of the disease, possibly leading to multisystemic improvement. The first is the DYNE-101 (by DYNE Therapeutics), a molecule composed of an antigen-binding fragment antibody (Fab) conjugated to an antisense oligonucleotide (ASO). Preclinical data showed a reduction in nuclear foci and splicing restoration in patient cells, knockdown of toxic human mRNA, and correction of splicing in a mouse model of MD1. DYNE-101 can improve myotonia after a single dose in mice. Finally, DYNE-101 showed a favorable safety profile and a significant reduction in wild-type DMPK RNA in non-human primates. The second product candidate is AOC 1001 (by AVIDITY Biosciences) a molecule composed of monoclonal antibodies binding the transferrin receptor 1 (TfR1) conjugated with a small interfering RNA (siRNA). In preclinical studies, AOC 1001 successfully delivered siRNAs to muscle cells, resulting in durable, dose-dependent reductions of DMPK RNA across a broad range of muscles including skeletal, cardiac, and smooth muscle. 

Both products are now completing Phase 1/2 clinical trials (ACHIEVE Clinical Trial for DYNE-101—NCT05481879, and MARINA Study for AOC 1001—NCT05027269) and an open-label extension study for the MARINA study (MARINA-OLE—NCT05027269, URL accessed on 20 December 2023—http://www.clinicaltrials.gov). 

## Figures and Tables

**Table 1:** Quality set measures proposed for MD1 patients from the Italian Neuro-Cardiology Network (INCN).

Quality Set Measures Proposed for MD1 Patients from the Italian Neuro-Cardiology Network (INCN)
MD1 Pharmacological Treatment
Administration of appropriate pharmacological therapy according to MD1 symptoms
MD1 Management
2. Multidisciplinary care plan developed or updated 3. Evaluation of cardiovascular status 4. Evaluation of neurological status 5. Evaluation of pulmonary, gastrointestinal, endocrinological, ophthalmological, gynecological/urological, or other status 6. Patient referred for physical, occupational, or speech/swallowing therapy 7. nutrition status or growth trajectories monitored 8. Patient queried about pain and pain interference with function
MD1 Planning and Patient Engagement
9. Patient counseled about advanced health care decision making, palliative care, or end of life issues
